# *PLOS ONE* 2014 Reviewer Thank You

**DOI:** 10.1371/journal.pone.0121093

**Published:** 2015-02-27

PLOS and the *PLOS ONE* editorial team would like to express our tremendous gratitude to all those individuals who participated in the peer review process this past year at *PLOS ONE*. 2014 was an amazing year for *PLOS ONE*; during the year, over 80,000 reviewers from around the world and across disciplines provided their expert input that led to publication of over 30,000 articles.

The names of our 2014 *PLOS ONE* reviewers are listed in S1 Reviewer List. It’s a lengthy list rivaled only by the depth of our gratitude. Thank you to all our reviewers for generously sharing your time, insight and expertise with *PLOS ONE* authors in the evaluation of their work. Your efforts are a key reason *PLOS ONE* is a successful publication and continues to be a force for positive change in scientific and medical publishing.

## Supporting Information

S1 Reviewer List Alphabetical list of 2014 *PLOS ONE* reviewers. (TXT)
